# New iterative criteria for strong $\mathcal{H}$-tensors and an application

**DOI:** 10.1186/s13660-017-1323-1

**Published:** 2017-02-27

**Authors:** Jingjing Cui, Guohua Peng, Quan Lu, Zhengge Huang

**Affiliations:** Department of Applied Mathematics, Northwestern Polytechnical University, Xi’an, Shaanxi 710072 P.R. China

**Keywords:** 15A18, 15A21, 15A69, strong $\mathcal{H}$-tensors, positive definiteness, irreducible, non-zero elements chain

## Abstract

Strong $\mathcal{H}$-tensors play an important role in identifying the positive definiteness of even-order real symmetric tensors. In this paper, some new iterative criteria for identifying strong $\mathcal{H}$-tensors are obtained. These criteria only depend on the elements of the tensors, and it can be more effective to determine whether a given tensor is a strong $\mathcal{H}$-tensor or not by increasing the number of iterations. Some numerical results show the feasibility and effectiveness of the algorithm.

## Introduction

A tensor can be regarded as a higher-order generalization of a matrix. Let $\mathbb{C}(\mathbb{R})$ denote the set of all complex (real) numbers and $N=\{1,2,\ldots,n\}$. We call $\mathcal {A}=(a_{i_{1}i_{2}\cdots i_{m}})$ an *m*th-order *n*-dimensional complex (real) tensor, if

$$a_{i_{1}i_{2}\cdots i_{m}}\in\mathbb{C}(\mathbb{R}), $$

where $i_{j}=1,2,\ldots,n$ for $j=1,2,\ldots,m$ [1, 2]. Obviously, a vector is a tensor of order 1 and a matrix is a tensor of order 2. A tensor $\mathcal {A}=(a_{i_{1}i_{2}\cdots i_{m}})$ is called symmetric [3], if

$$a_{i_{1}i_{2}\cdots i_{m}}=a_{\pi(i_{1}i_{2}\cdots i_{m})}, \quad\forall\pi \in\Pi_{m}, $$

where $\Pi_{m}$ is the permutation group of *m* indices. Furthermore, an *m*th-order *n*-dimensional tensor $\mathcal{I}=(\delta_{i_{1}i_{2}\cdots i_{m}})$ is called the unit tensor [4], if its entries

$$\delta_{i_{1}i_{2}\cdots i_{m}}= \textstyle\begin{cases} 1, & \mbox{if } i_{1}=\cdots=i_{m},\\ 0, & \mbox{otherwise}. \end{cases} $$

Let $\mathcal{A}=(a_{i_{1}i_{2}\cdots i_{m}})$ be an *m*th-order *n*-dimensional complex tensor. If there exist a number $\lambda\in\mathbb{C}$ and a non-zero vector $x=(x_{1},x_{2},\ldots ,x_{n})^{\mathrm{T}}\in\mathbb{C}^{n}$ that are solutions of the following homogeneous polynomial equations:

$$\mathcal{A}x^{m-1}=\lambda x^{[m-1]}, $$

then we call *λ* an eigenvalue of $\mathcal{A}$ and *x* the eigenvector of $\mathcal{A}$ associated with *λ* [1, 5–7], where $\mathcal{A}x^{m-1}$ and $\lambda x^{[m-1]}$ are vectors, whose *i*th components are

$$\bigl(\mathcal{A}x^{m-1}\bigr)_{i}=\sum _{i_{2},i_{3},\ldots,i_{m}\in N}a_{ii_{2}\cdots i_{m}}x_{i_{2}}\cdots x_{i_{m}} $$

and

$$\bigl(x^{[m-1]}\bigr)_{i}=x_{i}^{m-1}, $$

respectively. In particular, if *λ* and *x* are restricted in the real field, then we call *λ* an *H*-eigenvalue of $\mathcal{A}$ and *x* an *H*-eigenvector of $\mathcal{A}$ associated with *λ* [1].

In addition, the spectral radius of a tensor $\mathcal{A}$ is defined as

$$\rho(\mathcal{A})=\max\bigl\{ \vert \lambda \vert :\lambda \mbox{ is an eigenvalue of } \mathcal{A}\bigr\} . $$

Analogous with that of *M*-matrices, comparison matrices and *H*-matrices, the definitions of $\mathcal{M}$-tensors, comparison tensors and strong $\mathcal{H}$-tensors are given by the following.

### Definition 1.1

[8]

Let $\mathcal{A}=(a_{i_{1}i_{2}\cdots i_{m}})$ be an *m*th-order *n*-dimensional complex tensor. $\mathcal{A}$ is called an $\mathcal{M}$-tensor if there exist a non-negative tensor $\mathcal{B}$ and a positive real number $\eta\geq\rho(\mathcal{B})$ such that $\mathcal{A}=\eta\mathcal {I}-\mathcal{B}$. If $\eta>\rho(\mathcal{B})$, then $\mathcal{A}$ is called a strong $\mathcal{M}$-tensor.

### Definition 1.2

[9]

Let $\mathcal{A}=(a_{i_{1}i_{2}\cdots i_{m}})$ be an *m*th-order *n*-dimensional complex tensor. We call another tensor $\mathcal{M}(\mathcal{A})=(m_{i_{1}i_{2}\cdots i_{m}})$ as the comparison tensor of $\mathcal{A}$ if

$$m_{i_{1}i_{2}\cdots i_{m}}= \textstyle\begin{cases} \vert a_{i_{1}i_{2}\cdots i_{m}}\vert , & \mbox{if } (i_{2},i_{3},\ldots ,i_{m})=(i_{1},i_{1},\ldots,i_{1});\\ -\vert a_{i_{1}i_{2}\cdots i_{m}}\vert , & \mbox{if } (i_{2},i_{3},\ldots ,i_{m})\neq(i_{1},i_{1},\ldots,i_{1}). \end{cases} $$

### Definition 1.3

[10]

Let $\mathcal{A}=(a_{i_{1}i_{2}\cdots i_{m}})$ be an *m*th-order *n*-dimensional complex tensor. $\mathcal{A}$ is called a strong $\mathcal{H}$-tensor if there is a positive vector $x=(x_{1},x_{2},\ldots,x_{n})^{\mathrm{T}}\in\mathbb{R}^{n}$ such that

1.1 $$ \vert a_{ii\cdots i}\vert x_{i}^{m-1}> \mathop{\sum_{i_{2},i_{3},\ldots ,i_{m}\in N,}}_{\delta_{ii_{2}\cdots i_{m}}=0} \vert a_{ii_{2}\cdots i_{m}}\vert x_{i_{2}}\cdots x_{i_{m}},\quad\forall i\in N. $$

### Definition 1.4

[10]

Let $\mathcal{A}=(a_{i_{1}i_{2}\cdots i_{m}})$ be an *m*th-order *n*-dimensional complex tensor. $\mathcal{A}$ is called a diagonally dominant tensor if

1.2 $$ \vert a_{ii\cdots i}\vert \geq \mathop{\sum _{i_{2},i_{3},\ldots,i_{m}\in N,}}_{\delta_{ii_{2}\cdots i_{m}}=0} \vert a_{ii_{2}\cdots i_{m}}\vert , \quad \forall i\in N. $$

We call $\mathcal{A}$ a strictly diagonally dominant tensor if all strict inequalities in (1.2) hold.

### Definition 1.5

[4]

An *m*th-order *n*-dimensional complex tensor $\mathcal {A}=(a_{i_{1}i_{2}\cdots i_{m}})$ is called reducible, if there exists a nonempty proper index subset $I\subset N$ such that

$$a_{i_{1}i_{2}\cdots i_{m}}=0,\quad \forall i_{1}\in I, \forall i_{2},\ldots, i_{m}\notin I. $$

We call $\mathcal{A}$ irreducible if $\mathcal{A}$ is not reducible.

### Definition 1.6

[2]

Let $\mathcal{A}=(a_{i_{1}i_{2}\cdots i_{m}})$ be an *m*th-order *n*-dimensional tensor and a *n*-by-*n* matrix $X=(x_{ij})$ on mode-*k* is defined

$$(\mathcal{A}\times_{k} X)_{i_{1}\cdots j_{k}\cdots i_{m}}=\sum _{i_{k}=1}^{n}a_{i_{1}\cdots i_{k}\cdots i_{m}}x_{i_{k}j_{k}}. $$

According to Definition 1.6, we denote

$$\bigl(\mathcal{A}X^{m-1}\bigr):=\mathcal{A}\times_{2}X \times_{3}\cdots\times _{m}X. $$

Particularly, for $X=\operatorname{diag}(x_{1},x_{2},\ldots,x_{n})$, the product of the tensor $\mathcal{A}$ and the matrix *X* is given by

$$\mathcal{B}=(b_{i_{1}i_{2}\cdots i_{m}})=\mathcal{A}X^{m-1},\quad b_{i_{1}i_{2}\cdots i_{m}}=a_{i_{1}i_{2}\cdots i_{m}}x_{i_{2}}x_{i_{3}} \cdots x_{i_{m}}, i_{j}\in N, j\in\{1,2,\ldots,m\}. $$

### Definition 1.7

[2]

Let $\mathcal{A}=(a_{i_{1}i_{2}\cdots i_{m}})$ be an *m*th-order *n*-dimensional complex tensor. For some $i,j\in N$ ($i\neq j$), if there exist indices $k_{1},k_{2},\ldots,k_{r}$ with

$$\mathop{\mathop{\sum_{i_{2},i_{3},\ldots,i_{m}\in N,}}_{\delta _{k_{s}i_{2}\cdots i_{m}}=0,}}_{k_{s+1}\in\{i_{2},i_{3},\ldots,i_{m}\} } \vert a_{k_{s}i_{2} \cdots i_{m}} \vert \neq0,\quad s=0,1, \ldots,r, $$

where $k_{0}=i,k_{r+1}=j$, we call there is a non-zero elements chain from *i* to *j*.

For an *m*th degree homogeneous polynomial of *n* variables $f(x)$ is denoted as

1.3 $$ f(x)=\sum_{i_{1},i_{2},\ldots,i_{m}\in N}a_{i_{1}i_{2}\cdots i_{m}}x_{i_{1}}x_{i_{2}} \cdots x_{i_{m}}, $$

where $x=(x_{1},x_{2},\ldots,x_{n})^{\mathrm{T}}\in\mathbb{R}^{n}$. When *m* is even, $f(x)$ is called positive definite if

$$f(x)>0, \quad\mbox{for any } x\in\mathbb{R}^{n}, x\neq0. $$

The homogeneous polynomial $f(x)$ in (1.3) is equivalent to the tensor product of an *m*th-order *n*-dimensional symmetric tensor $\mathcal{A}$ and $x^{m}$ defined by [11]

1.4 $$ f(x)=\mathcal{A}x^{m}=\sum_{i_{1},i_{2},\ldots,i_{m}\in N}a_{i_{1}i_{2}\cdots i_{m}}x_{i_{1}}x_{i_{2}} \cdots x_{i_{m}}, $$

where $x=(x_{1},x_{2},\ldots,x_{n})^{\mathrm{T}}\in\mathbb{R}^{n}$. It is well known that the positive definiteness of multivariate polynomial $f(x)$ plays an important role in the stability study of nonlinear autonomous systems [8, 12]. For $n\leq3$, the positive definiteness of the multivariate polynomial form can be checked by a method based on the Sturm theorem [13]. However, for $n>3$ and $m\geq4$, it is difficult to determine a given even-order multivariate polynomial $f(x)$ is positive semi-definite or not because the problem is NP-hard. For solving this problem, Qi [1] pointed out that $f(x)$ defined by (1.4) is positive definite if and only if the real symmetric tensor $\mathcal{A}$ is positive definite, and provided an eigenvalue method to verify the positive definiteness of $\mathcal{A}$ when *m* is even (see Lemma 1.1).

### Lemma 1.1

[1]

*Let* $\mathcal{A}$ *be an even*-*order real symmetric tensor*, *then* $\mathcal{A}$ *is positive definite if and only if all of its H*-*eigenvalues are positive*.

Although from Lemma 1.1 we can verify the positive definiteness of an even-order symmetric tensor $\mathcal{A}$ (the positive definiteness of the *m*th-degree homogeneous polynomial $f(x)$) by computing the *H*-eigenvalues of $\mathcal{A}$. In [14–16], for a non-negative tensor, some algorithms are provided to compute its largest eigenvalue. And in [17, 18], based on semi-definite programming approximation schemes, some algorithms are also given to compute eigenvalues for general tensors with moderate sizes. However, it is difficult to compute all these *H*-eigenvalues when *m* and *n* are large. Recently, by introducing the definition of strong $\mathcal{H}$-tensor [9, 10], Li *et al.* [10] provided a practical sufficient condition for identifying the positive definiteness of an even-order symmetric tensor (see Lemma 1.2).

### Lemma 1.2

[10]

*Let* $\mathcal{A}=(a_{i_{1}i_{2}\cdots i_{m}})$ *be an even*-*order real symmetric tensor with* $a_{k\cdots k}>0$ *for all* $k\in N$. *If* $\mathcal {A}$ *is a strong* $\mathcal{H}$-*tensor*, *then* $\mathcal{A}$ *is positive definite*.

As mentioned in [19], it is still difficult to determine a strong $\mathcal{H}$-tensor in practice by using the definition of strong $\mathcal{H}$-tensor because the conditions ‘there is a positive vector $x=(x_{1},x_{2},\ldots,x_{n})^{\mathrm{T}}\in\mathbb {R}^{n}$ such that, for all $i\in N$, the Inequality (1.1) holds’ in Definition 1.3 is unverifiable for there are an infinite number of positive vector in $\mathbb{R}^{n}$. Therefore, much literature has focused on researching how to determine that a given tensor is a strong $\mathcal{H}$-tensor by using the elements of the tensors without Definition 1.3 recently, consequently, the corresponding even-order real symmetric tensor is positive definite. Therefore, the main aim of this paper is to study some new iterative criteria for identifying strong $\mathcal{H}$-tensors only depending on the elements of the tensors.

Before presenting our results, we review the existing ones that relate to the criteria for strong $\mathcal{H}$-tensors. Let *S* be an arbitrary nonempty subset of *N* and let $N\backslash S$ be the complement of *S* in *N*. Given an *m*th-order *n*-dimensional complex tensor $\mathcal{A}=(a_{i_{1}i_{2}\cdots i_{m}})$, we denote

$$\begin{aligned}& N^{m-1}=\{i_{2}i_{3}\cdots i_{m}: i_{j}\in N, j=2,3,\ldots ,m\}; \\& S^{m-1}=\{i_{2}i_{3}\cdots i_{m}: i_{j}\in S, j=2,3,\ldots,m\} ; \\& N^{m-1}\backslash S^{m-1}=\bigl\{ i_{2}i_{3} \cdots i_{m}: i_{2}i_{3}\cdots i_{m}\in N^{m-1} \mbox{ and } i_{2}i_{3}\cdots i_{m}\notin S^{m-1}\bigr\} ; \\& r_{i}(\mathcal{A})=\mathop{\sum_{i_{2},i_{3},\ldots,i_{m}\in N,}}_{\delta_{ii_{2}\cdots i_{m}}=0} \vert a_{ii_{2}\cdots i_{m}}\vert =\sum_{i_{2},i_{3},\ldots,i_{m}\in N} \vert a_{ii_{2}\cdots i_{m}}\vert -\vert a_{ii\cdots i}\vert ; \\& r_{i}^{j}(\mathcal{A})=\mathop{\sum _{\delta_{ii_{2}\cdots i_{m}}=0,}}_{\delta_{ji_{2}\cdots i_{m}}=0} \vert a_{ii_{2}\cdots i_{m}}\vert =r_{i}(\mathcal{A})-\vert a_{ij\cdots j}\vert ; \\& N_{1}=N_{1}(\mathcal{A})=\bigl\{ i\in N: \vert a_{ii\cdots i}\vert >r_{i}(\mathcal {A})\bigr\} ; \\& N_{2}=N_{2}(\mathcal{A})=\bigl\{ i\in N: 0< \vert a_{ii\cdots i}\vert \leq r_{i}(\mathcal{A})\bigr\} ; \\& s_{i}=\frac{|a_{ii\cdots i}|}{r_{i}(\mathcal{A})},\qquad t_{i}=\frac {r_{i}(\mathcal{A})}{|a_{ii\cdots i}|}, \qquad\overline{r}=\max\Bigl\{ \max_{i\in N_{2}}s_{i},\max _{i\in N_{1}}t_{i}\Bigr\} ; \\& r=\max_{i\in N_{1}} \biggl\{ \frac{\sum_{i_{2},i_{3},\ldots ,i_{m}\in N^{m-1} \backslash N_{1}^{m-1}}|a_{ii_{2}\cdots i_{m}}|}{|a_{ii\cdots i}|-\mathop{\sum_{i_{2},i_{3},\ldots ,i_{m}\in N_{1}^{m-1},}}_{\delta_{ii_{2}\cdots i_{m}}=0} |a_{ii_{2}\cdots i_{m}}|} \biggr\} ; \\& R_{i}^{(1)}(\mathcal{A})=\sum_{i_{2},i_{3},\ldots,i_{m}\in N^{m-1}\backslash N_{1}^{m-1}} \vert a_{ii_{2}\cdots i_{m}}\vert +r \sum_{\substack {i_{2},i_{3},\ldots,i_{m}\in N_{1}^{m-1},\\ \delta_{ii_{2}\cdots i_{m}}=0}}|a_{ii_{2}\cdots i_{m}}|,\quad \forall i\in N_{1}. \end{aligned}$$

In [10], Li *et al.* obtained the following result.

### Lemma 1.3

*Let* $\mathcal{A}=(a_{i_{1}i_{2}\cdots i_{m}})$ *be a complex tensor of order m dimension n*. *If there is an index* $i\in N$ *such that for all* $j\in N, j\neq i$,

$$\vert a_{ii\cdots i}\vert \bigl(\vert a_{jj\cdots j}\vert -r_{j}^{i}(\mathcal{A})\bigr)>r_{i}(\mathcal {A}) \vert a_{ji\cdots i}\vert , $$

*then* $\mathcal{A}$ *is a strong* $\mathcal{H}$-*tensor*.

In [20], Wang and Sun derived the following result.

### Lemma 1.4

*Let* $\mathcal{A}=(a_{i_{1}i_{2}\cdots i_{m}})$ *be an order m dimension n complex tensor*. *If*

$$\begin{aligned}& \vert a_{ii\cdots i}\vert s_{i}>\overline{r}\sum _{\substack{i_{2},i_{3},\ldots ,i_{m}\in N^{m-1}\backslash N_{1}^{m-1},\\ \delta_{ii_{2}\cdots i_{m}}=0}}\vert a_{ii_{2}\cdots i_{m}}\vert +\sum _{i_{2},i_{3},\ldots, i_{m}\in N_{1}}\max_{j\in\{i_{2},i_{3},\ldots,i_{m}\}}\{t_{j}\} \vert a_{ii_{2}\cdots i_{m}}\vert ,\quad \forall i\in N_{2}, \end{aligned}$$

*then* $\mathcal{A}$ *is a strong* $\mathcal{H}$-*tensor*.

Recently, Li *et al.* in [19] showed the following.

### Lemma 1.5

*Let* $\mathcal{A}=(a_{i_{1}i_{2}\cdots i_{m}})$ *be an order m dimension n complex tensor*. *If*

$$\begin{aligned}& \vert a_{ii\cdots i}\vert >\sum_{\substack{i_{2},i_{3},\ldots,i_{m}\in N^{m-1}\backslash N_{1}^{m-1},\\ \delta_{ii_{2}\cdots i_{m}}=0}}\vert a_{ii_{2}\cdots i_{m}}\vert +\sum_{i_{2},i_{3},\ldots, i_{m}\in N_{1}}\max _{j\in\{i_{2},i_{3},\ldots,i_{m}\}}\frac {r_{j}(\mathcal{A})}{|a_{jj\cdots j}|}\vert a_{ii_{2}\cdots i_{m}}\vert ,\quad \forall i\in N_{2}, \end{aligned}$$

*then* $\mathcal{A}$ *is a strong* $\mathcal{H}$-*tensor*.

In the sequel, Wang *et al.* in [21] proved the following result.

### Lemma 1.6

*Let* $\mathcal{A}=(a_{i_{1}i_{2}\cdots i_{m}})$ *be a complex tensor with order m and dimension n*. *If for all* $i\in N_{2}, j\in N_{1}$,

$$\begin{aligned}& \biggl(R_{j}^{(1)}(\mathcal{A})-\sum _{\substack{j_{2},j_{3},\ldots,j_{m}\in N_{1}^{m-1},\\ \delta_{jj_{2}\cdots j_{m}}=0}}\max_{k\in\{ j_{2},j_{3},\ldots,j_{m}\}}\frac{R_{k}^{(1)}(\mathcal{A})}{|a_{kk\cdots k}|}\vert a_{jj_{2}\cdots j_{m}}\vert \biggr)\\& \qquad {}\times \biggl(\vert a_{ii\cdots i}\vert -\sum _{\substack {i_{2},i_{3},\ldots,i_{m}\in N^{m-1}\backslash N_{1}^{m-1},\\ \delta _{ii_{2}\cdots i_{m}}=0}}\vert a_{ii_{2}\cdots i_{m}}\vert \biggr) \\& \quad>\sum_{t_{2},t_{3},\ldots,t_{m}\in N^{m-1}\backslash N_{1}^{m-1}} \vert a_{jt_{2}\cdots t_{m}}\vert \sum_{l_{2},l_{3},\ldots ,l_{m}\in N_{1}^{m-1}}\max_{l\in\{l_{2},l_{3},\ldots,l_{m}\} } \frac{R_{l}^{(1)}(\mathcal{A})}{|a_{ll\cdots l}|}\vert a_{il_{2}\cdots l_{m}}\vert , \end{aligned}$$

*then* $\mathcal{A}$ *is a strong* $\mathcal{H}$-*tensor*.

In this paper, we continue this research on criteria for strong $\mathcal{H}$-tensors; inspired by the ideas of [21], we obtain some new iterative criteria for strong $\mathcal{H}$-tensors, which improve the aforementioned Lemmas 1.3-1.6. As applications of the new iterative criteria for strong $\mathcal{H}$-tensors, we establish some sufficient conditions of the positive definiteness for an even-order real symmetric tensor. Numerical examples are implemented to illustrate these facts.

Now, some notations are given, which will be used throughout this paper. Let

$$\begin{aligned}& Z=\{0,1,2,\ldots\},\qquad h^{(0)}=r,\qquad\delta _{i}^{(0)}=1, \qquad\delta_{i}^{(1)}=\frac{R_{i}^{(1)}(\mathcal {A})}{|a_{ii\cdots i}|},\quad\forall i\in N_{1}; \\& h^{(1)}=\max_{i\in N_{1}} \biggl\{ \frac{\sum_{i_{2},i_{3},\ldots,i_{m}\in N^{m-1}\backslash N_{1}^{m-1}}|a_{ii_{2}\cdots i_{m}}|}{R_{i}^{(1)}(\mathcal{A})-\sum_{\substack{i_{2},i_{3},\ldots,i_{m}\in N_{1}^{m-1},\\ \delta _{ii_{2}\cdots i_{m}}=0}}\max_{j\in\{i_{2},i_{3},\ldots,i_{m}\} }\delta_{j}^{(1)}|a_{ii_{2}\cdots i_{m}}|} \biggr\} ; \\& R_{i}^{(l+1)}(\mathcal{A})=\sum_{i_{2},i_{3},\ldots,i_{m}\in N^{m-1}\backslash N_{1}^{m-1}} \vert a_{ii_{2}\cdots i_{m}}\vert \\& \phantom{R_{i}^{(l+1)}(\mathcal{A})=} {}+h^{(l)}\sum _{\substack{i_{2},i_{3},\ldots,i_{m}\in N_{1}^{m-1},\\ \delta _{ii_{2}\cdots i_{m}}=0}}\max_{j\in\{i_{2},i_{3},\ldots,i_{m}\} }\delta_{j}^{(l)} \vert a_{ii_{2}\cdots i_{m}}\vert ,\quad \forall i\in N_{1}, l\in Z; \\& \delta_{i}^{(l+1)}=\frac{R_{i}^{(l+1)}(\mathcal{A})}{|a_{ii\cdots i}|}, \quad\forall i\in N_{1}, l\in Z; \\& h^{(l+1)}=\max_{i\in N_{1}} \biggl\{ \frac{\sum_{i_{2},i_{3},\ldots,i_{m}\in N^{m-1}\backslash N_{1}^{m-1}}|a_{ii_{2}\cdots i_{m}}|}{R_{i}^{(l+1)}(\mathcal{A})-\sum_{\substack{i_{2},i_{3},\ldots,i_{m}\in N_{1}^{m-1},\\ \delta _{ii_{2}\cdots i_{m}}=0}}\max_{j\in\{i_{2},i_{3},\ldots,i_{m}\} }\delta_{j}^{(l+1)}|a_{ii_{2}\cdots i_{m}}|} \biggr\} ,\quad l\in Z. \end{aligned}$$

The remainder of the paper is organized as follows. In Section 2.1, some criteria for identifying strong $\mathcal{H}$-tensors are obtained; as an interesting application of these criteria, some sufficient conditions of the positive definiteness for an even-order real symmetric tensor are presented in Section 2.2. Numerical examples are given to verify the corresponding results. Finally, some conclusions are given to end this paper in Section 3.

We adopt the following notations throughout this paper. The calligraphy letters $\mathcal{A}, \mathcal{B}$, $\mathcal{H}, \ldots$ denote tensors; the capital letters $A, B, D, \ldots $ represent matrices; the lowercase letters $x, y,\ldots $ refer to vectors.

## Main results

### Criteria for identifying strong $\mathcal{H}$-tensors

In this subsection, we give some new criteria for identifying strong $\mathcal{H}$-tensors by making use of elements of tensors only. For the convenience of our discussion, we start with the following lemmas, which will be useful in the next proofs.

#### Lemma 2.1

*Let* $\mathcal{A}=(a_{i_{1}i_{2}\cdots i_{m}})$ *be an* *mth*-*order* *n*-*dimensional complex tensor*, *then*, *for all* $i\in N_{1}, l=1,2,\ldots$ ,

- $1\geq h^{(l)}\geq0$;
- $1>\delta_{i}^{(1)}\geq h^{(1)}\delta_{i}^{(1)}\geq\delta _{i}^{(2)}\geq\cdots\geq\delta_{i}^{(l)}\geq h^{(l)}\delta_{i}^{(l)}\geq \delta_{i}^{(l+1)}\geq\cdots\geq0$.

#### Proof

Since $i\in N_{1}$, we have $0\leq r<1$. Moreover, for $i\in N_{1}$, we get

$$\begin{aligned}& r\geq\frac{\sum_{i_{2},i_{3},\ldots,i_{m}\in N^{m-1} \backslash N_{1}^{m-1}}|a_{ii_{2}\cdots i_{m}}|}{|a_{ii\cdots i}|-\sum_{\substack{i_{2},i_{3},\ldots,i_{m}\in N_{1}^{m-1},\\ \delta _{ii_{2}\cdots i_{m}}=0}}|a_{ii_{2}\cdots i_{m}}|}, \quad \vert a_{ii\cdots i}\vert -\sum _{\substack{i_{2},i_{3},\ldots,i_{m}\in N_{1}^{m-1},\\ \delta_{ii_{2}\cdots i_{m}}=0}}\vert a_{ii_{2}\cdots i_{m}}\vert >0, \end{aligned}$$

which implies

$$\begin{aligned}& r\vert a_{ii\cdots i}\vert \geq\sum_{i_{2},i_{3},\ldots,i_{m}\in N^{m-1} \backslash N_{1}^{m-1}} \vert a_{ii_{2}\cdots i_{m}}\vert +r\sum_{\substack {i_{2},i_{3},\ldots,i_{m}\in N_{1}^{m-1},\\ \delta_{ii_{2}\cdots i_{m}}=0}}\vert a_{ii_{2}\cdots i_{m}}\vert =R_{i}^{(1)}(\mathcal{A}). \end{aligned}$$

From the above inequality, $\forall i\in N_{1}$, we obtain

$$0 \leq\delta_{i}^{(1)}=\frac{R_{i}^{(1)}(A)}{|a_{ii\cdots i}|}\leq r< 1. $$

Together with the expression of $R_{i}^{(1)}(\mathcal{A})$, for $\forall i\in N_{1}$, we deduce that

$$\begin{aligned}& \frac{\sum_{i_{2},i_{3},\ldots,i_{m}\in N^{m-1}\backslash N_{1}^{m-1}}|a_{ii_{2}\cdots i_{m}}|}{R_{i}^{(1)}(\mathcal{A})-\sum_{\substack{i_{2},i_{3},\ldots,i_{m}\in N_{1}^{m-1},\\ \delta _{ii_{2}\cdots i_{m}}=0}}\max_{j\in\{i_{2},i_{3},\ldots,i_{m}\} }\delta_{j}^{(1)}|a_{ii_{2}\cdots i_{m}}|}\\& \quad =\frac{R_{i}^{(1)}(\mathcal{A})-r\sum_{\substack {i_{2},i_{3},\ldots,i_{m}\in N^{m-1},\\ \delta_{ii_{2}\cdots i_{m}}=0}}|a_{ii_{2}\cdots i_{m}}|}{R_{i}^{(1)}(\mathcal{A})-\sum_{\substack{i_{2},i_{3},\ldots,i_{m}\in N_{1}^{m-1},\\ \delta _{ii_{2}\cdots i_{m}}=0}}\max_{j\in\{i_{2},i_{3},\ldots,i_{m}\} }\delta_{j}^{(1)}|a_{ii_{2}\cdots i_{m}}|}\leq1. \end{aligned}$$

Combining the expression of $h^{(1)}$ and the above inequality results in

2.1 $$ 0\leq h^{(1)} \leq1. $$

Besides, for $\forall i\in N_{1}$,

$$\begin{aligned}& R_{i}^{(1)}(\mathcal{A})=\sum_{i_{2},i_{3},\ldots,i_{m}\in N^{m-1}\backslash N_{1}^{m-1}} \vert a_{ii_{2}\cdots i_{m}}\vert +r\sum_{\substack {i_{2},i_{3},\ldots,i_{m}\in N_{1}^{m-1},\\ \delta_{ii_{2}\cdots i_{m}}=0}}\vert a_{ii_{2}\cdots i_{m}}\vert \leq r_{i}(\mathcal{A})< \vert a_{i\cdots i}\vert , \end{aligned}$$

that is,

2.2 $$ \delta_{i}^{(1)}=\frac{R_{i}^{(1)}(\mathcal{A})}{|a_{ii\cdots i}|}\leq \frac{r_{i}(\mathcal{A})}{|a_{ii\cdots i}|}< 1. $$

Since

$$\begin{aligned}& h^{(1)}=\max_{i\in N_{1}} \biggl\{ \frac{\sum_{i_{2},i_{3},\ldots,i_{m}\in N^{m-1}\backslash N_{1}^{m-1}}|a_{ii_{2}\cdots i_{m}}|}{R_{i}^{(1)}(\mathcal{A})-\sum_{\substack{i_{2},i_{3},\ldots,i_{m}\in N_{1}^{m-1},\\ \delta _{ii_{2}\cdots i_{m}}=0}}\max_{j\in\{i_{2},i_{3},\ldots,i_{m}\} }\delta_{j}^{(1)}|a_{ii_{2}\cdots i_{m}}|} \biggr\} , \end{aligned}$$

for $\forall i\in N_{1}$, we have

$$\begin{aligned}& h^{(1)}\geq\frac{\sum_{i_{2},i_{3},\ldots,i_{m}\in N^{m-1}\backslash N_{1}^{m-1}}|a_{ii_{2}\cdots i_{m}}|}{R_{i}^{(1)}(\mathcal{A})-\sum_{\substack {i_{2},i_{3},\ldots,i_{m}\in N_{1}^{m-1},\\ \delta_{ii_{2}\cdots i_{m}}=0}}\max_{j\in\{i_{2},i_{3},\ldots,i_{m}\}}\delta _{j}^{(1)}|a_{ii_{2}\cdots i_{m}}|}, \end{aligned}$$

which entails

$$\begin{aligned} h^{(1)}R_{i}^{(1)}(\mathcal{A}) \geq & \sum _{i_{2},i_{3},\ldots ,i_{m}\in N^{m-1}\backslash N_{1}^{m-1}}\vert a_{ii_{2}\cdots i_{m}}\vert + h^{(1)}\sum _{\substack{i_{2},i_{3},\ldots,i_{m}\in N_{1}^{m-1},\\ \delta_{ii_{2}\cdots i_{m}}=0}}\max_{j\in\{ i_{2},i_{3},\ldots,i_{m}\}} \delta_{j}^{(1)}\vert a_{ii_{2}\cdots i_{m}}\vert \\ =& R_{i}^{(2)}(\mathcal{A}). \end{aligned}$$

Dividing by $|a_{ii\cdots i}|$ on both sides of the above inequality yields

2.3 $$ h^{(1)}\delta_{i}^{(1)}=h^{(1)} \frac{R_{i}^{(1)}(\mathcal {A})}{|a_{ii\cdots i}|}\geq\frac{R_{i}^{(2)}(\mathcal {A})}{|a_{ii\cdots i}|}=\delta_{i}^{(2)}. $$

For $i\in N_{1}$, it follows from (2.1)-(2.3) that

$$\begin{aligned}& 1>\delta_{i}^{(1)}\geq h^{(1)}\delta_{i}^{(1)} \geq\delta_{i}^{(2)}\geq 0. \end{aligned}$$

Furthermore, by the expression of $R_{i}^{(2)}(\mathcal{A})$ and the above inequality, for $i\in N_{1}$, we have

$$\begin{aligned}& \frac{\sum_{i_{2},i_{3},\ldots,i_{m}\in N^{m-1}\backslash N_{1}^{m-1}}|a_{ii_{2}\cdots i_{m}}|}{R_{i}^{(2)}(\mathcal{A})-\sum_{\substack{i_{2},i_{3},\ldots,i_{m}\in N_{1}^{m-1},\\ \delta _{ii_{2}\cdots i_{m}}=0}}\max_{j\in\{i_{2},i_{3},\ldots,i_{m}\} }\delta_{j}^{(2)}|a_{ii_{2}\cdots i_{m}}|} \\& \quad=\frac{R_{i}^{(2)}(\mathcal{A})-h^{(1)}\sum_{\substack {i_{2},i_{3},\ldots,i_{m}\in N_{1}^{m-1},\\ \delta_{ii_{2}\cdots i_{m}}=0}}\max_{j\in\{i_{2},i_{3},\ldots,i_{m}\}}\delta _{j}^{(1)}|a_{ii_{2}\cdots i_{m}}|}{R_{i}^{(2)}(\mathcal{A})-\sum_{\substack{i_{2},i_{3},\ldots,i_{m}\in N_{1}^{m-1},\\ \delta _{ii_{2}\cdots i_{m}}=0}}\max_{j\in\{i_{2},i_{3},\ldots,i_{m}\} }\delta_{j}^{(2)}|a_{ii_{2}\cdots i_{m}}|}\leq1. \end{aligned}$$

Combining the expression of $h^{(2)}$ and the above inequality results in

2.4 $$ 0\leq h^{(2)} \leq1. $$

In the same manner as applied in the proof of (2.3), for $i\in N_{1}$, we obtain

2.5 $$ h^{(2)}\delta_{i}^{(2)}\geq \delta_{i}^{(3)}. $$

For $i\in N_{1}$, it follows from inequalities (2.4) and (2.5) that

$$\delta_{i}^{(2)}\geq h^{(2)}\delta_{i}^{(2)} \geq\delta_{i}^{(3)}\geq 0. $$

By an analogical proof as above, we can derive that, for $i\in N_{1}, l=3,4,\ldots$ ,

$$\begin{aligned}& 1\geq h^{(l)}\geq0; \\& \delta_{i}^{(2)}\geq h^{(2)}\delta_{i}^{(2)} \geq\delta_{i}^{(3)}\geq h^{(3)}\delta_{i}^{(3)} \geq\cdots\geq\delta_{i}^{(l+1)}\geq h^{(l+1)} \delta_{i}^{(l+1)}\geq\delta_{i}^{(l+2)}\geq \cdots\geq 0. \end{aligned}$$

The proof is completed. □

#### Lemma 2.2

[10]

*If* $\mathcal{A}$ *is a strictly diagonally dominant tensor*, *then* $\mathcal{A}$ *is a strong* $\mathcal{H}$-*tensor*.

#### Lemma 2.3

[10]

*Let* $\mathcal{A}=(a_{i_{1}i_{2}\cdots i_{m}})$ *be an* *mth*-*order* *n*-*dimensional complex tensor*. *If* $\mathcal{A}$ *is a strong* $\mathcal {H}$-*tensor*, *then* $N_{1}\neq\emptyset$.

By Lemma 2.2, if $N_{2}=\emptyset$ ($\mathcal{A}$ is a strictly diagonally dominant tensor), then $\mathcal{A}$ is a strong $\mathcal {H}$-tensor; by Lemma 2.3, if $\mathcal{A}$ is a strong $\mathcal{H}$-tensor, then $N_{1}\neq\emptyset$. Hence, we always assume that $N_{1}\neq\emptyset, N_{2}\neq\emptyset$. In addition, we also assume that $\mathcal{A}$ satisfies $a_{ii\cdots i}\neq0, r_{i}(\mathcal{A})\neq0, \forall i\in N$.

#### Lemma 2.4

[10]

*Let* $\mathcal{A}=(a_{i_{1}i_{2}\cdots i_{m}})$ *be an* *mth*-*order* *n*-*dimensional complex tensor*. *If* $\mathcal{A}$ *is irreducible*,

$$\vert a_{ii\cdots i}\vert \geq r_{i}(\mathcal{A}), \quad\forall i\in N, $$

*and strictly inequality holds for at least one* *i*, *then* $\mathcal{A}$ *is a strong* $\mathcal{H}$-*tensor*.

#### Lemma 2.5

[10]

*Let* $\mathcal{A}=(a_{i_{1}i_{2}\cdots i_{m}})$ *be an* *mth*-*order* *n*-*dimensional tensor*. *If there exists a positive diagonal matrix* *X* *such that* $AX^{m-1}$ *is a strong* $\mathcal{H}$-*tensor*, *then* $\mathcal{A}$ *is a strong* $\mathcal{H}$-*tensor*.

#### Lemma 2.6

[22]

*Let* $\mathcal{A}=(a_{i_{1}i_{2}\cdots i_{m}})$ *be an* *mth*-*order* *n*-*dimensional complex tensor*. *If*

(i) $|a_{ii\cdots i}|\geq r_{i}(\mathcal{A})$, $\forall i\in N$,
(ii) $N_{1}= \{i\in N: |a_{ii\cdots i}|> r_{i}(\mathcal{A}) \} \neq\emptyset$,
(iii) *for any* $i\notin N_{1}$, *there exists a non*-*zero elements chain from* *i* *to* *j* *such that* $j\in N_{1}$,

*then* $\mathcal{A}$ *is a strong* $\mathcal{H}$-*tensor*.

#### Theorem 2.1

*Let* $\mathcal{A}=(a_{i_{1}i_{2}\cdots i_{m}})$ *be an* *mth*-*order* *n*-*dimensional complex tensor*. *If there exists* $l\in Z$ *such that*

2.6 $$\begin{aligned} \vert a_{ii\cdots i}\vert > & h^{(l+1)}\sum _{i_{2},i_{3},\ldots,i_{m}\in N_{1}^{m-1}}\max_{j\in\{i_{2},i_{3},\ldots,i_{m}\}}\delta _{j}^{(l+1)} \vert a_{ii_{2}\cdots i_{m}}\vert \\ &{}+\sum_{\substack {i_{2},i_{3},\ldots,i_{m}\in N^{m-1}\backslash N_{1}^{m-1},\\ \delta _{ii_{2}\cdots i_{m}}=0}}\vert a_{ii_{2}\cdots i_{m}}\vert ,\quad \forall i\in N_{2}, \end{aligned}$$

*then* $\mathcal{A}$ *is a strong* $\mathcal{H}$-*tensor*.

#### Proof

By the expression of $h^{(l+1)}$, it follows that

$$\begin{aligned}& h^{(l+1)}\geq\frac{\sum_{i_{2},i_{3},\ldots,i_{m}\in N^{m-1}\backslash N_{1}^{m-1}}|a_{ii_{2}\cdots i_{m}}|}{R_{i}^{(l+1)}(\mathcal{A})-\sum_{\substack {i_{2},i_{3},\ldots,i_{m}\in N_{1}^{m-1},\\ \delta_{ii_{2}\cdots i_{m}}=0}}\max_{j\in\{i_{2},i_{3},\ldots,i_{m}\}}\delta _{j}^{(l+1)}|a_{ii_{2}\cdots i_{m}}|}, \quad\forall i\in N_{1}, \end{aligned}$$

equivalently,

2.7 $$\begin{aligned} h^{(l+1)}R_{i}^{(l+1)}(\mathcal{A}) \geq & \sum_{i_{2},i_{3},\ldots ,i_{m}\in N^{m-1}\backslash N_{1}^{m-1}}\vert a_{ii_{2}\cdots i_{m}}\vert \\ &{}+h^{(l+1)}\sum_{\substack{i_{2},i_{3},\ldots,i_{m}\in N_{1}^{m-1},\\ \delta_{ii_{2}\cdots i_{m}}=0}}\max_{j\in\{ i_{2},i_{3},\ldots,i_{m}\}} \delta_{j}^{(l+1)}\vert a_{ii_{2}\cdots i_{m}}\vert . \end{aligned}$$

From Lemma 2.1, we have

$$\begin{aligned}& 0\leq h^{(l+1)}\delta_{i}^{(l+1)}< 1, \quad\forall i\in N_{1}. \end{aligned}$$

Together with Inequality (2.6), there exists a $\varepsilon>0$, sufficiently small such that for all $i\in N_{1}$,

2.8 $$ 0< h^{(l+1)}\delta_{i}^{(l+1)}+ \varepsilon< 1, $$

and for all $i\in N_{2}$,

2.9 $$\begin{aligned}& \vert a_{ii\cdots i}\vert -h^{(l+1)}\sum _{i_{2},i_{3},\ldots,i_{m}\in N_{1}^{m-1}}\max_{j\in\{i_{2},i_{3},\ldots,i_{m}\}}\delta _{j}^{(l+1)} \vert a_{ii_{2}\cdots i_{m}}\vert -\sum_{\substack {i_{2},i_{3},\ldots,i_{m}\in N^{m-1}\backslash N_{1}^{m-1},\\ \delta _{ii_{2}\cdots i_{m}}=0}}\vert a_{ii_{2}\cdots i_{m}}\vert \\& \quad>\varepsilon\sum_{i_{2},i_{3},\ldots,i_{m}\in N_{1}^{m-1}}\vert a_{ii_{2}\cdots i_{m}}\vert . \end{aligned}$$

Let the matrix $X = \operatorname{diag}(x_{1},x_{2},\ldots,x_{n})$, where

$$x_{i}= \textstyle\begin{cases} (h^{(l+1)}\delta_{i}^{(l+1)}+\varepsilon)^{\frac{1}{m-1}}, & i\in N_{1};\\ 1,& i\in N_{2}. \end{cases} $$

We see by Inequality (2.8) that $(h^{(l+1)}\delta _{i}^{(l+1)}+\varepsilon)^{\frac{1}{m-1}}<1$ ($\forall i\in N_{1}$), as $\varepsilon\neq\infty, x_{i}\neq\infty$, which shows that *X* is a diagonal matrix with positive entries. Let $\mathcal{B}=\mathcal{A}X^{m-1}$. Next, we will prove that $\mathcal{B}$ is strictly diagonally dominant.

For any $i\in N_{1}$, it follows from (2.7) that

$$\begin{aligned} r_{i}(\mathcal{B}) \leq&\sum_{\substack {i_{2},i_{3},\ldots,i_{m}\in N_{1}^{m-1},\\ \delta_{ii_{2}\cdots i_{m}}=0}}\vert a_{ii_{2}\cdots i_{m}}\vert \bigl(h^{(l+1)}\delta _{i_{2}}^{(l+1)}+ \varepsilon\bigr)^{\frac{1}{m-1}}\cdots\bigl(h^{(l+1)}\delta _{i_{m}}^{(l+1)}+\varepsilon\bigr)^{\frac{1}{m-1}}\\ &{}+\sum _{i_{2},i_{3},\ldots,i_{m}\in N^{m-1}\backslash N_{1}^{m-1}}\vert a_{ii_{2}\cdots i_{m}}\vert \\ \leq& \sum_{\substack{i_{2},i_{3},\ldots,i_{m}\in N_{1}^{m-1},\\ \delta_{ii_{2}\cdots i_{m}}=0}}\vert a_{ii_{2}\cdots i_{m}}\vert \Bigl(h^{(l+1)}\max_{j\in\{i_{2},i_{3},\ldots,i_{m}\}}\delta _{j}^{(l+1)}+ \varepsilon\Bigr)\\ &{}+\sum_{i_{2},i_{3},\ldots,i_{m}\in N^{m-1}\backslash N_{1}^{m-1}}\vert a_{ii_{2}\cdots i_{m}} \vert \\ \leq&\varepsilon\sum_{\substack{i_{2},i_{3},\ldots,i_{m}\in N_{1}^{m-1},\\ \delta_{ii_{2}\cdots i_{m}}=0}}\vert a_{ii_{2}\cdots i_{m}} \vert +h^{(l+1)}R_{i}^{(l+1)}(\mathcal{A}) \\ < & \varepsilon \vert a_{ii\cdots i}\vert +h^{(l+1)}R_{i}^{(l+1)}( \mathcal {A}) \\ =& \vert a_{ii\cdots i}\vert \bigl(\varepsilon+h^{(l+1)} \delta_{i}^{(l+1)}\bigr) \\ =& \vert b_{ii\cdots i}\vert . \end{aligned}$$

For any $i\in N_{2}$, it follows from (2.9) that

$$\begin{aligned} r_{i}(\mathcal{B}) \leq&\sum_{i_{2},i_{3},\ldots ,i_{m}\in N_{1}^{m-1}}\vert a_{ii_{2}\cdots i_{m}}\vert \bigl( h^{(l+1)}\delta _{i_{2}}^{(l+1)}+ \varepsilon\bigr)^{\frac{1}{m-1}}\cdots\bigl( h^{(l+1)}\delta _{i_{m}}^{(l+1)}+\varepsilon\bigr)^{\frac{1}{m-1}}\\ &{}+\sum _{\substack {i_{2},i_{3},\ldots,i_{m}\in N^{m-1}\backslash N_{1}^{m-1},\\ \delta _{ii_{2}\cdots i_{m}}=0}}\vert a_{ii_{2}\cdots i_{m}}\vert \\ \leq&\sum_{i_{2},i_{3},\ldots,i_{m}\in N_{1}^{m-1}}\vert a_{ii_{2}\cdots i_{m}}\vert \Bigl( h^{(l+1)}\max_{j\in\{ i_{2},i_{3},\ldots,i_{m}\}}\delta_{j}^{(l+1)}+ \varepsilon\Bigr)\\ &{}+\sum_{\substack{i_{2},i_{3},\ldots, i_{m}\in N^{m-1}\backslash N_{1}^{m-1},\\ \delta_{ii_{2}\cdots i_{m}}=0}}\vert a_{ii_{2}\cdots i_{m}} \vert \\ < & \vert a_{ii\cdots i}\vert =\vert b_{ii\cdots i}\vert . \end{aligned}$$

Therefore, from the above inequalities, we conclude that $|b_{ii\cdots i}|>r_{i}(\mathcal{B})$ for all $i\in N$, $\mathcal{B}$ is strictly diagonally dominant, and by Lemma 2.2, $\mathcal{B}$ is a strong $\mathcal{H}$-tensor. Further, by Lemma 2.5, $\mathcal{A}$ is a strong $\mathcal{H}$-tensor. □

#### Remark 2.1

If $N_{1}$ contains only one element, then Theorem 2.1 reduces to Lemma 1.3, and if $l=0$, then Theorem 2.1 reduces to Lemma 1.6.

#### Theorem 2.2

*Let* $\mathcal{A}=(a_{i_{1}i_{2}\cdots i_{m}})$ *be an* *mth*-*order* *n*-*dimensional complex tensor*. *If* $\mathcal{A}$ *is irreducible and there exists* $l\in Z$ *such that for all* $i\in N_{2}$

2.10 $$ \vert a_{ii\cdots i}\vert \geq h^{(l+1)}\sum _{i_{2},i_{3},\ldots,i_{m}\in N_{1}^{m-1}}\max_{j\in\{i_{2},i_{3},\ldots,i_{m}\}}\delta _{j}^{(l+1)} \vert a_{ii_{2}\cdots i_{m}}\vert +\sum_{\substack {i_{2},i_{3},\ldots,i_{m}\in N^{m-1}\backslash N_{1}^{m-1},\\ \delta _{ii_{2}\cdots i_{m}}=0}}\vert a_{ii_{2}\cdots i_{m}}\vert , $$

*in addition*, *the strict inequality holds for at least one* $i\in N_{2}$, *then* $\mathcal{A}$ *is a strong* $\mathcal{H}$-*tensor*.

#### Proof

Notice that $\mathcal{A}$ is irreducible; this implies

$$\sum_{i_{2},i_{3},\ldots,i_{m}\in N^{m-1}\backslash N_{1}^{m-1}} \vert a_{ii_{2}\cdots i_{m}}\vert >0, \quad i\in N_{1}. $$

Let the matrix $X = \operatorname{diag}(x_{1},x_{2},\ldots,x_{n})$, where

$$x_{i}= \textstyle\begin{cases} (h^{(l+1)}\delta_{i}^{(l+1)})^{\frac{1}{m-1}}, & i\in N_{1};\\ 1, & i\in N_{2}. \end{cases} $$

Adopting the same procedure as in the proof of Theorem 2.1, we conclude that $|b_{i\cdots i}|\geq r_{i}(\mathcal{B})$ for all $i\in N$. Moreover, the strict inequality holds for at least one $i\in N_{2}$, thus, there exists at least an $i\in N$ such that $|b_{ii\cdots i}|>r_{i}(\mathcal{B})$.

On the other hand, since $\mathcal{A}$ is irreducible, and so is $\mathcal{B}$. Then by Lemma 2.4, we see that $\mathcal{B}$ is a strong $\mathcal{H}$-tensor. By Lemma 2.5, $\mathcal{A}$ is also a strong $\mathcal{H}$-tensor. □

#### Remark 2.2

If $l=0$, then Theorem 2.2 reduces to Theorem 2.6 of [21].

Let

$$\begin{aligned} J =& \biggl\{ i\in N_{2}: \vert a_{ii\cdots i}\vert >h^{(l+1)}\sum_{i_{2},i_{3},\ldots,i_{m}\in N_{1}^{m-1}}\max_{j\in\{ i_{2},i_{3},\ldots,i_{m}\}} \delta_{j}^{(l+1)}\vert a_{ii_{2}\cdots i_{m}}\vert \\ &{}+\sum _{\substack{i_{2},i_{3},\ldots,i_{m}\in N^{m-1}\backslash N_{1}^{m-1},\\ \delta_{ii_{2}\cdots i_{m}}=0}}\vert a_{ii_{2}\cdots i_{m}}\vert \biggr\} . \end{aligned}$$

#### Theorem 2.3

*Let* $\mathcal{A}=(a_{i_{1}i_{2}\cdots i_{m}})$ *be an* *mth*-*order* *n*-*dimensional tensor*. *If for all* $i\in N_{2}$

$$\begin{aligned}& \vert a_{ii\cdots i}\vert \geq h^{(l+1)}\sum _{i_{2},i_{3},\ldots,i_{m}\in N_{1}^{m-1}}\max_{j\in\{i_{2},i_{3},\ldots,i_{m}\}}\delta _{j}^{(l+1)} \vert a_{ii_{2}\cdots i_{m}}\vert +\sum_{\substack {i_{2},i_{3},\ldots,i_{m}\in N^{m-1}\backslash N_{1}^{m-1},\\ \delta _{ii_{2}\cdots i_{m}}=0}}\vert a_{ii_{2}\cdots i_{m}}\vert , \end{aligned}$$

*and if* $\forall i\in N\backslash J\neq\emptyset$, *there exists a non*-*zero elements chain from* *i* *to* *j* *such that* $j\in J\neq\emptyset $, *then* $\mathcal{A}$ *is a strong* $\mathcal{H}$-*tensor*.

#### Proof

Let the matrix $X = \operatorname{diag}(x_{1},x_{2},\ldots,x_{n})$, where

$$\begin{aligned}& x_{i}= \textstyle\begin{cases} (h^{(l+1)}\delta_{i}^{(l+1)})^{\frac{1}{m-1}}, & i\in N_{1};\\ 1, & i\in N_{2}. \end{cases}\displaystyle \end{aligned}$$

Similar to the proof of Theorem 2.1, we can obtain $|b_{ii\cdots i}|\geq r_{i}(\mathcal{B})$ for all $i\in N$, and there exists at least an $i\in N_{2}$ such that $|b_{ii\cdots i}|>r_{i}(\mathcal{B})$.

On the other hand, if $|b_{ii\cdots i}|=r_{i}(\mathcal{B})$, then $i\in N\backslash J$; by the assumption, we know that there exists a non-zero elements chain of $\mathcal{A}$ from *i* to *j*, such that $j\in J$. Then there exists a non-zero elements chain of $\mathcal{B}$ from *i* to *j*, such that *j* satisfies $|b_{jj\cdots j}|>r_{j}(\mathcal{B})$.

Based on the above analysis, we conclude that the tensor $\mathcal{B}$ satisfies the conditions of Lemma 2.6, so $\mathcal{B}$ is a strong $\mathcal{H}$-tensor. By Lemma 2.5, $\mathcal{A}$ is a strong $\mathcal {H}$-tensor. The proof is completed. □

#### Remark 2.3

If $l=0$, then Theorem 2.3 reduces to Theorem 2.7 of [21].

#### Remark 2.4

From Lemma 2.1, we can also obtain smaller iterative coefficients $h^{(l+1)}\delta_{i}^{(l+1)}$ by increasing *l*. Therefore, Theorem 2.1 in this paper can be more effective to determine whether a given tensor is a strong $\mathcal{H}$-tensor or not by increasing the number of iterations.

#### Example 2.1

Consider a tensor $\mathcal{A}=(a_{ijk})$ with 3-order and 4-dimension defined as follows:

$$\begin{aligned}& \mathcal{A}=\bigl[A(1,:,:),A(2,:,:),A(3,:,:),A(4,:,:)\bigr], \\& A(1,:,:)=\left ( \begin{matrix} 15.5 & 0.5 & 0.5 & 0 \\ 0.5 & 20 & 1 & 0 \\ 0 & \frac{5}{6} & 0.5 & 0.5 \\ 0 & 0.5 & 0.5 & 0.5 \end{matrix} \right ),\qquad A(2,:,:)=\left ( \begin{matrix} 1 & 0 & 0.5 & 0.5 \\ 0.5 & 12 & 0 & 0.5 \\ 1 & 0.5 & 0.5 & 0.5 \\ 1 & 0.5 & 0.5 & 0.5 \end{matrix} \right ), \\& A(3,:,:)=\left ( \begin{matrix} 1 & 1 & 0& 0.5 \\ 0 & 0.5 & 0.5 & 0.5 \\ 0 & 0 & 8 & 0 \\ 0.5 & 0.5 & 0 & 1 \end{matrix} \right ),\qquad A(4,:,:)=\left ( \begin{matrix} 0.5 & 0.5 & 1 & 0 \\ 0.5 & 1 & 0 & 0.5 \\ 0.5 & 0 & 1 & 0 \\ 1 & 1 & 0.5 & 10 \end{matrix} \right ). \end{aligned}$$

Obviously,

$$\begin{aligned}& \vert a_{111}\vert =15.5, \qquad r_{1}(\mathcal{A})= \frac{155}{6},\qquad \vert a_{222}\vert =12, \qquad r_{2}(\mathcal{A})=8, \\& \vert a_{333}\vert =8, \qquad r_{3}(\mathcal{A})=6, \qquad \vert a_{444}\vert =10, \qquad r_{4}( \mathcal{A})=8, \end{aligned}$$

so $N_{1}(\mathcal{A})=\{2,3,4\}, N_{2}(\mathcal{A})=\{1\}$. First of all, it can be verified that Lemmas 1.3-1.6 cannot determine whether the tensor $\mathcal{A}$ is a strong $\mathcal{H}$-tensor or not. However, Theorem 2.1 in this paper can verify that the tensor $\mathcal{A}$ is a strong $\mathcal{H}$-tensor when $l=1$.

In fact, by Lemma 1.3,

$$\vert a_{333}\vert \bigl(\vert a_{111}\vert -r_{1}^{3}\bigr)=-78.6667< 3=r_{3}\vert a_{133}\vert , $$

by Lemma 1.4, $\overline{r}=\max\{s_{1},\max_{i\in N_{2}}t_{i}\} =\max\{\frac{r_{1}(\mathcal{A})}{a_{111}}, \max_{i\in N_{2}}\frac {a_{iii}}{r_{i}(\mathcal{A})}\}=0.8$,

$$\vert a_{111}\vert s_{1}=9.3< 17.8833=\overline{r}\sum _{\substack {i_{2},i_{3}\in N^{2}\backslash N_{1}^{2},\\ \delta _{1i_{2}i_{3}}=0}}\vert a_{1i_{2}i_{3}}\vert +\sum _{i_{2},i_{3}\in N_{1}^{2}}\max_{j\in\{i_{2},i_{3}\}}\{t_{j}\} \vert a_{1i_{2}i_{3}}\vert , $$

by Lemma 1.5,

$$\vert a_{111}\vert =15.5< 18.1833=\sum _{\substack{i_{2},i_{3}\in N^{2}\backslash N_{1}^{2},\\ \delta _{1i_{2}i_{3}}=0}}\vert a_{1i_{2}i_{3}}\vert +\sum _{i_{2},i_{3}\in N_{1}^{2}}\max_{j\in\{i_{2},i_{3}\}}\frac{r_{j}(\mathcal {A})}{|a_{jjj}|}\vert a_{1i_{2}i_{3}}\vert , $$

and, by Lemma 1.6,

$$\begin{aligned}& \biggl(R_{2}^{(1)}(\mathcal{A})-\sum _{\substack{i_{2},i_{3}\in N_{1}^{2},\\ \delta_{2i_{2}i_{3}}=0}}\max_{j\in\{i_{2},i_{3}\} }\frac{R_{j}^{(1)}(\mathcal {A})}{|a_{jjj}|}\vert a_{2i_{2}i_{3}}\vert \biggr) \biggl(\vert a_{111}\vert -\sum _{\substack {i_{2},i_{3}\in N^{2}\backslash N_{1}^{2},\\ \delta _{1i_{2}i_{3}}=0}}\vert a_{1i_{2}i_{3}}\vert \biggr) \\& \quad=4.5417\times14=63.5838 \\& \quad< 63.8127=\sum_{i_{2},i_{3}\in N^{2}\backslash N_{1}^{2}}\vert a_{2i_{2}i_{3}} \vert \sum_{i_{2},i_{3}\in N_{1}^{2}}\max_{j\in\{i_{2},i_{3}\}} \frac{R_{j}^{(1)}(\mathcal {A})}{|a_{jjj}|}\vert a_{1i_{2}i_{3}}\vert . \end{aligned}$$

However, by calculation with Matlab 7.11.0, $r=0.667$ and the results of $R_{i}^{(l+1)}(\mathcal{A}), \delta_{i}^{(l+1)}, h^{(l+1)}$ ($i\in\{ 2,3,4\}$) of Theorem 2.1 in this paper are given in Table 1 for the total number of iterations $l=4$. When $l=1$, we can get

$$\begin{aligned}& \vert a_{111}\vert =15.5>15.4300=h^{(2)}\sum _{i_{2},i_{3}\in N_{1}^{2}}\max_{j\in\{i_{2},i_{3}\}}\delta_{j}^{(2)} \vert a_{1i_{2} i_{3}}\vert + \sum_{\substack{i_{2},i_{3}\in N^{2}\backslash N_{1}^{2},\\ \delta _{1i_{2}i_{3}}=0}}\vert a_{1i_{2}i_{3}}\vert , \end{aligned}$$

we see that $\mathcal{A}$ satisfies the conditions of Theorem 2.1, then $\mathcal{A}$ is a strong $\mathcal{H}$-tensor. In fact, there exists a positive diagonal matrix $X=\operatorname{diag}(1,0.7489,0.7812,0.7978)$ such that $\mathcal{A}X^{2}$ is strictly diagonally dominant.

**Table 1:** **The results of** $\pmb{R_{i}^{(l+1)}(\mathcal{A})}$ **and** $\pmb{\delta _{i}^{(l+1)}}$ **and** $\pmb{h^{(l+1)}}$ **(** $\pmb{i\in\{2,3,4\}}$ **)**

***l***	$\boldsymbol {R_{2}^{(l+1)}(\mathcal{A})}$	$\boldsymbol {R_{3}^{(l+1)}(\mathcal{A})}$	$\boldsymbol {R_{4}^{(l+1)}(\mathcal{A})}$	$\boldsymbol {\delta_{2}^{(l+1)}}$	$\boldsymbol {\delta _{3}^{(l+1)}}$	$\boldsymbol {\delta_{4}^{(l+1)}}$	$\boldsymbol {h^{(l+1)}}$
0	6.8333	5.000	6.6667	0.5694	0.6250	0.6667	0.9908
1	6.7706	4.9128	6.5046	0.5642	0.6141	0.6505	0.9937
2	6.7261	4.8782	6.4636	0.5605	0.6098	0.6464	0.9999
3	6.7255	4.8777	6.4628	0.5605	0.6097	0.6463	1.0000
4	6.7254	4.8776	6.4627	0.5604	0.6097	0.6463	1.0000

### An application: the positive definiteness of an even-order real symmetric tensor

In this subsection, by making use of the results in Section 2.1, we present new criteria for identifying the positive definiteness of an even-order real symmetric tensor.

From Lemma 1.2 and Theorems 2.1-2.3, we easily obtain the following result.

#### Theorem 2.4

*Let* $\mathcal{A}=(a_{i_{1}i_{2}\cdots i_{m}})$ *be an even*-*order real symmetric tensor with* *mth*-*order* *n*-*dimension*, *and* $a_{i\cdots i}>0$ *for all* $i\in N$. *If* $\mathcal{A}$ *satisfies one of the following conditions*:

(i) *all the conditions of Theorem* 2.1;
(ii) *all the conditions of Theorem* 2.2;
(iii) *all the conditions of Theorem* 2.3,

*then* $\mathcal{A}$ *is positive definite*.

#### Example 2.2

Let

$$\begin{aligned} f(x) =& \mathcal {A}x^{4}=16x_{1}^{4}+21x_{2}^{4}+23x_{3}^{4}+19x_{4}^{4}-8x_{1}^{3}x_{4}+12x_{1}^{2}x_{2}x_{3}\\ &{}-12x_{2}x_{3}^{2}x_{4} +4x_{2}x_{4}^{3}+4x_{3}x_{4}^{3}-24x_{1}x_{2}x_{3}x_{4} \end{aligned}$$

be a 4th-degree homogeneous polynomial. We can get an 4th-order 4-dimensional real symmetric tensor $\mathcal {A}=(a_{i_{1}i_{2}i_{3}i_{4}})$, where

$$\begin{aligned}& a_{1111}=16, \qquad a_{2222}=21, \qquad a_{3333}=23, \qquad a_{4444}=19, \\& a_{1114}=a_{1141}=a_{1411}=a_{4111}=-2, \\& a_{2444}=a_{4244}=a_{4424}=a_{4442}=1, \\& a_{3444}=a_{4344}=a_{4434}=a_{4443}=1, \\& a_{1123}=a_{1132}=a_{1213}=a_{1312}=a_{1231}=a_{1321}=1, \\& a_{2113}=a_{2131}=a_{2311}=a_{3112}=a_{3121}=a_{3211}=1, \\& a_{3234}=a_{3243}=a_{3324}=a_{3342}=a_{3423}=a_{3432}=-1, \\& a_{2334}=a_{2343}=a_{2433}=a_{4233}=a_{4323}=a_{4332}=-1, \\& a_{1234}=a_{1243}=a_{1324}=a_{1342}=a_{1423}=a_{1432}=-1, \\& a_{2134}=a_{2143}=a_{2314}=a_{2341}=a_{2413}=a_{2431}=-1, \\& a_{3124}=a_{3142}=a_{3214}=a_{3241}=a_{3412}=a_{3421}=-1, \\& a_{4123}=a_{4132}=a_{4213}=a_{4231}=a_{4312}=a_{4321}=-1, \end{aligned}$$

and other $a_{i_{1}i_{2}i_{3}i_{4}}=0$. By calculation, we have

$$\vert a_{1111}\vert =16< 18=r_{1}(\mathcal{A}) $$

and

$$\begin{aligned}& \vert a_{2222}\vert \bigl(a_{1111}-r_{1}( \mathcal{A})+\vert a_{1222}\vert \bigr)=-42< 0=r_{2}( \mathcal {A})\vert a_{1222}\vert . \end{aligned}$$

Hence, $\mathcal{A}$ is not a strictly diagonally dominant tensor defined in [23], or a quasi-doubly strictly diagonally dominant tensor defined in [22], so we cannot use Theorem 3 in [23] and Theorem 4 in [22] to identify the positive definiteness of $\mathcal{A}$. Further, it can be verified that $\mathcal{A}$ satisfies all the conditions of Theorem 2.1. Thus, from Theorem 2.4, we can see that $\mathcal{A}$ is positive definite, that is, $f(x)$ is positive definite. In fact, there exists a positive diagonal matrix $X=\operatorname{diag}(1,0.8110,0.8243,0.8043)$ such that $\mathcal{A}X^{3}$ is strictly diagonally dominant. Therefore, $\mathcal {A}$ is a strong $\mathcal{H}$-tensor.

## Conclusions

In this paper, we give some criteria for identifying a strong $\mathcal {H}$-tensor which only depend on the elements of tensors, and by increasing the number of iterations, we can determine whether a given tensor is a strong $\mathcal{H}$-tensor or not more effective. We also present new criteria for identifying the positive definiteness of an even-order real symmetric tensor based on these criteria.
